# Figurative language processing in atypical populations: the ASD perspective

**DOI:** 10.3389/fnhum.2015.00024

**Published:** 2015-02-17

**Authors:** Mila Vulchanova, David Saldaña, Sobh Chahboun, Valentin Vulchanov

**Affiliations:** ^1^Language Acquisition and Language Processing Lab, Department of Language and Literature, Norwegian University of Science and TechnologyTrondheim, Norway; ^2^Individual Differences, Language and Cognition Lab, Department of Developmental and Educational Psychology, University of SevilleSeville, Spain

**Keywords:** figurative language, autism spectrum disorders, metaphors, idioms, impaired processing mechanisms

## Abstract

This paper is intended to provide a critical overview of experimental and clinical research documenting problems in figurative language processing in atypical populations with a focus on the Autistic Spectrum. Research in the comprehension and processing of figurative language in autism invariably documents problems in this area. The greater paradox is that even at the higher end of the spectrum or in the cases of linguistically talented individuals with Asperger syndrome, where structural language competence is intact, problems with extended language persist. If we assume that figurative and extended uses of language essentially depend on the perception and processing of more concrete core concepts and phenomena, the commonly observed failure in atypical populations to understand figurative language remains a puzzle. Various accounts have been offered to explain this issue, ranging from linking potential failure directly to overall structural language competence (Norbury, 2005; Brock et al., 2008) to right-hemispheric involvement (Gold and Faust, 2010). We argue that the dissociation between structural language and figurative language competence in autism should be sought in more general cognitive mechanisms and traits in the autistic phenotype (e.g., in terms of weak central coherence, Vulchanova et al., 2012b), as well as failure at on-line semantic integration with increased complexity and diversity of the stimuli (Coulson and Van Petten, 2002). This perspective is even more compelling in light of similar problems in a number of conditions, including both acquired (e.g., Aphasia) and developmental disorders (Williams Syndrome). This dissociation argues against a simple continuity view of language interpretation.

## Introduction

Figurative language is a cover term for linguistic expressions whose interpretation is nonliteral, where the meaning of the expression as a whole cannot be computed directly from the meaning of its constituents. Figurative language can vary in types, degrees of extension from the literal and degrees of transparency, and structure. Moreover, figurative expressions can fluctuate from a single word to a long sentence. Here belong a range of phenomena, such as metaphors, idioms, proverbs, humor and jokes, hyperbole, indirect requests, clichés (Gibbs, 1999). Such expressions are characterized by interpretations which cannot be retrieved by simply knowing the basic senses of the constituent lexical items, and where the addressee needs to arrive at the intended meaning rather than what is being said literally.

It has been claimed that it is exactly the need to go beyond the literal interpretation and grasp the intended meaning that makes figurative language special and more demanding for processing (Levorato and Cacciari, 2002). Unlike literal language, such expressions depend more heavily on both linguistic and visual context, and are often—in fact, impossible—to understand in the absence of such context. Still, in everyday communication much of the meaning is implied, and can be understood following linguistic and contextual cues (Coulson, 2005). It is this context sensitivity of natural language that has inspired the continuity claim that figurative language is not exceptional. From this perspective, all language and all its sentences are multiply ambiguous whereby the content of all utterances largely underdetermines their interpretation (Gibbs, 1994; Sperber and Wilson, 2006). This approach suggests that figurative language is rather to be found on a continuum from literal-to loose-to metaphorical language and should not be considered as a departure from normal language use. While this is one of the more radical interpretations, all approaches arguing for a lack of exceptionality in figurative language sustain that it is pervasive both in language and in thought (Fauconnier, 1997, 2007; Lakoff and Johnson, 1980; Lakoff, 1987; Turner, 1991). If this is true, then it is not a special form of language.

Yet, research in developmental disorders documents subtle dissociations between the ability to understand literal expressions and the comprehension of nonliteral (figurative) language. For instance, high-functioning individuals with autism with intact structural language skills often fail to understand the meaning of jokes, irony, and idiomatic language (Gold and Faust, 2010; Vulchanova et al., 2012a,b). Thus, they present a case against a simple continuum view of figurative language.

In this paper we present evidence from studies of figurative language processing in autism arguing that this evidence calls for a revision of a simple continuum view. We first review issues of relevance to our main topic, such as how to best approach and understand the similarities and differences in the processing of literal and figurative language. For this purpose we start by discussing evidence from typically developing children and adults, to then move on to comment on the data that can be found in looking at a population of special interest to figurative language, namely individuals with autism. We conclude by suggesting possible ways in which these data can be interpreted in the light of current cognitive accounts of autism and more broader approaches to language comprehension.

## Figurative language in typical populations

### Figurative language acquisition in typical development

Language development provides evidence of the somewhat different status of figurative language. It takes more time for children to begin to appreciate extended uses. According to Nippold (2006), the development of skills in processing metaphors, idioms and proverbs is an important part of semantic development. Compared to vocabulary acquisition and basic semantic skills, skills in the domain of figurative language emerge later. Thus, recent research (Levorato and Cacciari, 1995; Nippold, 1998, 2006; Kempler et al., 1999; Nippold and Duthie, 2003; Cain et al., 2009) suggests that the acquisition of idioms takes longer than vocabulary acquisition, and that it gradually takes off after age five and on.

Opinions, and findings, however, divide concerning the path of this development. Nippold (1998, 2006) and Nippold and Duthie (2003) assume that this is a gradual development, not essentially different from other lexical development, and that it continues also in adulthood. However, Kempler et al. (1999) show that the understanding of idioms follows a non-linear path, very similar to the vocabulary burst between the second and the third year (Marchman and Bates, 1994; Bates and Goodman, 1997). Unlike vocabulary, however, with idioms, this process takes approximately four times longer, with a peak at around 11 years (Vulchanova et al., 2011). In a study of 6- and 9-year-old children and adults, Laval and Bernicot (2002) provide evidence that only at age 9 can children start to appreciate and use context in idiom comprehension. Furthermore, only from this age on children show sensitivity to frequency and familiarity.

The appreciation of figurative language in development requires coordination between cognitive, linguistic and pragmatic skills (Tolchinsky, 2004; Bernicot et al., 2007). Several factors play a role in the acquisition and comprehension of figurative language. Among the most salient ones for idioms, for instance, are frequency of the expression, transparency of its structure, the context in which it is encountered, and linguistic skills and competences (Nippold and Duthie, 2003). It is commonly agreed and has been demonstrated that metalinguistic awareness facilitates the understanding of figurative language, including idioms (Levorato and Cacciari, 2002; Nippold and Duthie, 2003; Nippold, 2006). It has also been shown that reading comprehension is a strong predictor of idiom comprehension (Levorato et al., 2004).

Bernicot et al. (2007) investigated the order of acquisition of different types of nonliteral language in children. They studied the relationship between the children’s understanding and their meta-pragmatic competence, defined as the ability to distinguish between what is being said and what is meant in indirect language. In that study they looked at three different types of expressions: indirect requests, idioms and conversational implicatures, in a story completion task. Their results demonstrate that mastering advanced language skills and competencies, such as those required for figurative language processing, correlates with age. This may be attributed to the maturity needed for the processing of expressions offering increased complexity of the inference between the literal meaning (what we say) and the figurative one (what we mean).

### Accounts of figurative language processing

#### Metaphors and literal language

Metaphors are by far the most “popular” paradigmatic example of figurative language. At the level of thought, conceptual metaphor is a cognitive process by which we represent an abstract concept in terms of a more concrete and tangible one. Metaphors establish (novel) links or mappings between mental domains or spaces, typically a source one and a target one (Fauconnier, 1985). As such, they are ways of thinking capturing generalizations about the world around us and our experience of it.

Theories of metaphors differ in how they assume metaphors are processed, and whether they consider them a departure from normal (literal) language or not. The standard pragmatic view assumes that metaphors are expressions processed via mechanisms different from those used for literal meanings. On this view, the literal meaning should be accessed and rejected before arriving at the intended (figurative) meaning. This implies that an inference is necessary to access the appropriate intended meaning (Grice, 1975). Many authors consider metaphors as “special” structures which are present in everyday language and change depending on time and culture (Lakoff and Johnson, 1980; Turner, 1991).

Alternatively it is suggested that metaphorical and literal meanings are processed in parallel and also use the same mechanisms (Gibbs, 1994). Thus, for both lexical items and metaphorical language, processing interacts with information retrieved from the context (Gibbs, 1987, 1994). Gibbs et al. (1997) found that metaphors did not require more time to process than literal expressions. Furthermore, reaction times did not differ when the context was adequate. An important caveat here is, that equivalent processing times need not reflect equivalent effort (Coulson and Van Petten, 2002; Bambini and Resta, 2012).

Coulson and Van Petten (2002) point to evidence from processing studies suggesting that metaphoric language places heavier demands on processing and requires additional effort for alignment and inference than literal language, not in the least by placing additional demands on working memory (Blasko, 1999). They further argue that the continuity claim should be distinguished from the equivalence claim, which assumes that metaphoric language is no more difficult to comprehend than literal language. They adopt a conceptual blending approach to metaphor (Fauconnier and Turner, 1998), which explains metaphor comprehension as a dynamic process, which creates a blending space combining attributes from the source and target domains. Thus, interpretation arises as a result of selecting relevant properties of these domains and inhibiting aspects which are not relevant through a process of constant updating. In an ERP study, which tested three types of expressions, sentences that ended with words used literally, metaphorically and in an intermediate literal mapping condition, they document that metaphors elicit the greatest N400 effect, while literal mappings occupy a place between true metaphors and literal statements judging by brain responses. This study thus provides evidence for the continuity claim, and, at the same time, shows that metaphors are indeed more demanding for processing, but in a gradient way. The authors suggest that metaphor “taxes” the system we use to understand figurative meaning for two basic reasons. On the one hand, one needs to establish a mapping between elements in distantly related domains (e.g., unlike metonymy), and on the other, to retrieve information from memory to integrate these elements. Other studies have shown similar results (Pynte et al., 1996).

A common problem in assessing results from research in figurative language processing, as observed in Pickering and Frisson (2001), is that word frequency, plausibility, and cloze probability have not always been controlled in several studies reporting reading times for literal and figurative language. Such variables should be taken into account and would have produced different results when determining whether figurative language is more demanding compared to literal language.

Links between metaphors and other types of figurative expressions have been suggested. Gibbs (2003) argues that idioms, often considered as “dead” metaphors, in fact, offer a more dynamic metaphor-based processing. In another study, Gibbs et al. (1997) conducted a series of experiments using a priming method to investigate the role of conceptual metaphors in immediate idiom integration. The aim of the study was to establish whether conceptual metaphors were accessed faster in the context of idioms in discourse. Participants accessed conceptual metaphors more often for the purpose of understanding an idiom, and less so when they were processing literal expressions or literal paraphrases of idioms. Furthermore, this study demonstrated that people access the appropriate conceptual metaphor when they are integrating each specific idiom, and not a similar one with the same figurative meaning. This suggests that idioms with the same figurative meanings may be associated with different conceptual metaphors.

#### Processing of idiomatic expressions

As a form of nonliteral language, idioms have attracted attention both in theoretical linguistics and in empirical psycholinguistic research, as a result of their specific nature, both in terms of structure and organization. Unlike regular phrases and expressions, idioms come largely in a “pre-packaged” form, with many, if not all of their components which cannot be freely replaced or supplemented. Idioms are expressions of varying degree of frozenness and semantic transparency. On the one hand, they are retrieved from the lexicon because they have to be acquired and stored like lexical items, and, on the other, they are processed like structures generated by grammar (Jackendoff, 2002; Vulchanova et al., 2011). Due to this “double” nature or different levels of processing, understanding idioms may pose problems.

There are two kinds of theories regarding how idioms are processed and understood. According to the Lexical Representation hypothesis, idioms are stored as lexical items, and understanding an idiom involves two parallel processes, a retrieval process (which is faster), and a literal compositional computation process based on decomposing every element separately (Swinney and Cutler, 1979).

Hamblin and Gibbs (1999) highlight idiom decomposability and suggest that idiom interpretation depends on identifying the individual constituents, because most idioms are decomposable. It is thus suggested that the processing and understanding idioms cannot be reduced to lexical access or lexical retrieval only (Cacciari and Tabossi, 1988; Gibbs, 1992; Vega-Moreno, 2001). This type of approach bridges over to the second type of approach, the configuration hypothesis. Authors that support it assume that idioms are represented in a distributed way and they are processed as complex expressions (Cacciari and Tabossi, 1988). Tabossi et al. (2005) found that spoken idiom identification differs from word recognition. This means that the modality of presenting the idiom may affect the way we process these expressions as well.

A central question in all of the above approaches to idiom processing, but also more broadly to figurative language processing, is whether literal meanings are accessed first, and whether at all. Some authors reject the existence of literal or default meanings altogether (Sperber and Wilson, 2006), while others suggest a revision of the concept of literal meaning (Ariel, 2002). Recent experimental research, however, provides evidence of the existence of literal meaning, and support for a possible distinction between basic/literal senses and interpretations, and extended/derived ones. Foraker and Murphy (2012) investigated how polysemous senses are processed during sentence comprehension. In this study, in a condition where the context was neutral and did not bias towards a specific sense, participants read disambiguating sentences faster when these sentences were compatible with the dominant sense of target words. Rubio Fernandez (2007) provides further evidence of the “lingering” presence of literal meaning in the processing of figurative language in the domain of metaphors, where core features of word meaning remained activated even after the metaphorical meaning was retrieved.

Idioms are easier to understand in the presence of supportive context. It has been commonly established that the main role of context is to provide semantic support for decoding the target (appropriate) meaning of a sentence or an expression (Cacciari and Levorato, 1989; Gibbs, 1991; Levorato and Cacciari, 1995; Vega-Moreno, 2001; Laval, 2003).

Several factors influence idiom comprehension. Idiomatic expressions can vary in transparency. It is much easier to understand more transparent expressions that opaque ones. Another factor is familiarity. It is a variable that influences comprehension, and many studies establish that a higher degree of familiarity increases performance, leading to better results in different comprehension tasks (Gibbs, 1991; Levorato and Cacciari, 1995; Nippold and Taylor, 2002; Lacroix et al., 2010).

We suggest here that competence in figurative language is characterized by the ability to process language beyond the literal interpretation of individual words. This competency relies both on inferencing skills and on the ability to integrate contextual information from both verbal and nonverbal sources. We expand this idea further in the next sections.

While much of extended language use goes unnoticed to typical native speakers of a language, and, as such may appear part of normal communication, it may pose severe problems for children and adults with developmental deficits. Such populations offer a glimpse into subtle dissociations between literal and nonliteral (figurative) language. For instance, in the autistic spectrum, even high-functioning individuals are often described as overly literal and often fail to appreciate figurative expressions. Such dissociations speak against the view that there exist no basic senses of lexical items (Sperber and Wilson, 2006), since these senses appear to be the only ones available to these individuals. This if often displayed in problems in the autistic spectrum with resolving linguistic ambiguity. We devote the rest of this paper to analyzing this issue and reviewing what data from individuals with autism can tell us about the true nature of figurative language.

## Figurative language in autism spectrum disorder

Autism spectrum disorder (ASD) is a disorder characterized by impairments in social interaction and communication, and restricted behavior and interests. The impairment in social interaction can be manifested in marked deficiencies in the use of eye contact, reading facial expressions, emotions, body posture, and gestures. Failure to develop peer relationships, lack of spontaneous seeking to share enjoyment, interests, or achievements with other people and lack of social or emotional reciprocity, are also typical in autism.

Regarding the impairments in communication, the most common problem, even in individuals with adequate structural language, is the inability to initiate or sustain a conversation with others, including inability to maintain a topic shared with the interlocutor. In addition, ASD individuals frequently have a very stereotyped and repetitive use of language, thus leaving no room for spontaneity.

Individuals with ASD also show other restricted repetitive and stereotyped patterns of behavior, interests and activities. They may present encompassing preoccupation with one or more restricted patterns of interest. They are often characterized by lack of flexibility and adherence to specific, nonfunctional routines or rituals, repetitive motor mannerisms (e.g., hand or finger flapping or twisting, or complex whole-body movements). Often they display persistent preoccupation with parts of objects (e.g., wheels of a toy car).

### Poor figurative language in ASD

In typical language development, the acquisition of metalinguistic skills and the comprehension of figurative language seem to be achieved in childhood by the age of nine or ten years according to several authors. However, in ASD this process is typically delayed and depends on various factors, such as degree of language impairment, chronological age, context or social environment. Findings in research suggest that there is a delayed rate of development with regard to processing of ambiguity, idioms, metaphors and other types of figurative language in individuals with autism, and problems at more global levels of language structure, although performance may improve with age (Melogno et al., 2012a,b; Vulchanova et al., 2012a,b).

ASD is a disorder that significantly affects language and communication, and many individuals with ASD do not develop fluent language due to comorbidity with other impairments, such as intellectual disability or language disorder (LD). When LD is comorbid with autism, there are serious difficulties in understanding ambiguous linguistic information, as would be expected. In contrast, individuals with high-functioning autism are distinguished by relative preservation of linguistic and cognitive skills. They usually display a level of intelligence which is normal or even above average, and quite often have specific talents in certain areas. However, problems with pragmatic language skills have also been reported in their case, even with clear strengths in areas of grammar (Landa, 2000; Volden et al., 2009; Vulchanova et al., 2012a,b). Such dissociations in ASD between literal and figurative language argue against a simple continuity model.

One of the first studies to address figurative language comprehension and its roots in autism was the study by Happé (1995). It compared three groups of children with autism to a group of age- and VIQ-matched controls on the understanding of three types of expressions, synonyms, similes, and metaphors. In order to test the hypothesis that metaphor comprehension correlates with the ability to read minds and co-locutors’ intentions, participants were tested on both first-order, and second-order Theory-of-Mind (ToM) tasks. This study was inspired by ideas from Relevance Theory (Sperber and Wilson, 1986) and the aim was to put the basic assumptions of this account to the test, by investigating its predictions on the well-known problems in aspects of pragmatic language in autism. The basic idea with the language stimuli used in the study, administered as a sentence completion task, was that there is a gradation of difficulty in processing language, ranging from full transparency in literal expressions to close to full transparency (similes), to nontransparency (metaphors). The findings from this study confirm that metaphor comprehension is impaired in children and adolescents with autism, against adequate processing of similes, and that the ability to process metaphors is directly linked to ToM ability. Thus, the ASD participants who passed both first- and second-order ToM tasks outperformed both participants who solved only the first-order tasks and those who did not pass either task.

The ToM account of the well-attested problems in autism with metaphor comprehension was tested further by Norbury (2005). In her study, an alternative hypothesis was put forth, namely that language competence is a better predictor of performance on metaphor tasks. For this purpose Norbury tested ASD children first grouped according to language ability and autistic symptomatology, and in a second analysis on their ToM ability. Both types of groups were compared to typically developing age-matched peers. The study included a number of tasks to establish language status: the British Picture Vocabulary scales (BPVS; Dunn et al., 1997), the Concepts and Directions subtest of CELF-III (Semel et al., 2000), and the Recalling sentences subtest of CELF-III. Semantic knowledge was tested on the Test of Word Knowledge (ToWK; Wiig and Secord, 1992), which includes synonyms, figurative language interpretation (idiomatic phrases), word definitions and word ambiguity (polysemy) testing. The results of this study demonstrate that semantic ability, which is a core language skill, is a better predictor of metaphor comprehension, whereas ToM, even though it predicts a proportion of the variance, is a weaker predictor of figurative language processing. Thus, only the children with a language impairment, with and without autism, showed impaired metaphor understanding. Furthermore, first-order ToM skills, while probably necessary, are not sufficient to ensure success with figurative language interpretation. These results, however, should be interpreted with caution, since the test used to assess semantic knowledge (ToWK) includes a number of subtests assessing figurative language comprehension, and as such, should, by definition, predict performance on metaphorical expressions (Rundblad and Annaz, 2010).

While the above two studies have only investigated metaphors, further research has targeted a broader range of figurative expressions. MacKay and Shaw (2004) report performance on six categories of expressions, including hyperbole, indirect requests, irony, metonymy, rhetorical questions and understatement. The assumption in their design is that all of these categories involve interpreting what is intended, rather than said, in each expression. For this purpose, the authors used two measures, correct understanding of the meaning of the expression, and correct understanding of the intent of the speaker. In this study language stimuli were accompanied by supporting picture material to provide visual focus for the participants. The experimental group included high-functioning ASD children compared to a control group with no communicative difficulties. In addition to the statistical analysis of results, the study also includes many examples of children’s responses illustrating the specific pattern evident in the autistic spectrum in interpreting figurative expressions. This study documents a scale of difficulty in the area of figurative language, with irony standing out as (the most) challenging task, even for typical children, especially processing the intent of the speaker, and fewer problems in rhetorical questions, even for autistic children, where the meaning of such questions is accessible. Areas where a significant difference was observed between the typically developing children and the ASD group, include indirect requests (intent), understatements (intent), as well as metonymy (both meaning and intent). The latter category was problematic even in the presence of visual support cues, and especially when the visual cues were less suggestive. Based on the finding that ASD children performed at the same level as the control group on understanding the meaning of certain figurative expressions (indirect requests, rhetorical questions, understatement), but failed at understanding the intent of those same expressions, the authors suggest that this result may be caused by different levels of language competencies and skills in the two groups, not evident in the results on the vocabulary scales (BPVS). Unfortunately, this study cannot be compared to the above two, since it did not address metaphors (but only metonymy), and did not ask the same question, namely the extent to which figurative language interpretation depends on ToM ability. Yet, it establishes a scale of difficulty in the processing of indirect language and compares performance by ASD children to typically developing peers in a range of figurative expressions.

Whyte et al. (2014) more recently studied idioms in children with ASD ages 5–12 years. They tested them on idiom comprehension, advanced ToM, vocabulary, and syntax. Like the other studies on figurative language, they also found that they performed worse than children matched on chronological-age. They were not, however, worse at understanding idioms than a syntax-matched control group of younger children. These results would support Norbury (2005) view that language impairment is actually the strongest factor in predicting performance on figurative language tasks.

Beyond group studies, a couple of case studies have addressed figurative language in autism. Melogno et al. (2012a) provide a case study of two high-functioning ASD children. Participants were assessed twice, first prior to, and subsequently following an intervention. Even though, initially the two ASD children showed performance comparable to the average range of typical controls, their patterns of response were different. Assessment after intervention revealed improvement, but in a different way for each participant. Moreover, the level of performance was still below their chronological age, indicative of a “drift” in figurative language comprehension.

In our own work we have addressed the cognitive and language profiles of two high-functioning (Asperger) children with a talent for language learning (Vulchanova et al., 2012a,b). These two case studies tested, among other language competencies, idiom comprehension and metaphor comprehension. Both participants in the studies displayed a highly deviant profile in idiom knowledge compared to similarly-aged controls. In the younger participant, the gap in performance with chronological age was huge (*z* = −3.08). Moreover, the participant performed poorer even than much younger children on the same task, suggesting a deviant developmental trajectory. Even though the gap with chronological age was somewhat smaller for the older participant (*z* = −2.22), it was still significant. The same participant showed an atypical pattern of responses to metaphorical expressions based on the design by Gold et al. (2010). In contrast to typical age-matched controls, for this participant, reaction times to novel metaphors and nonsense expressions were similar, reflecting a problem in distinguishing between these two types of expressions and assessing their plausibility.

Some studies have failed to find significant differences in accuracy scores for participants with autism in studies of figurative language comprehension. For example, Colich et al. (2012) tested whether children and adolescents with autism were able to interpret the ironic intent of speakers. Although both typically developing and autistic participants showed longer response times to ironic comments, brain activation profile was more bilateral in the case of the ASD group, indicating a potential compensation mechanism in processing this kind of figurative language. The study by Pexman et al. (2011) also found similar responses in an irony comprehension task, with results in eye-tracking variables and judgment latencies indicating that individuals with autism might be using different mechanisms to respond.

### Developmental aspects of figurative language in ASD

In addition to the role of structural language abilities, a great deal of research has explored the influence of other variables that could potentially explain this deficit in the interpretation of nonliteral meaning in participants with autism. In this sense, an interesting question is whether these skills develop in relation to chronological age also in this population, and further whether there is development in relation to mental age. This was first studied from a developmental perspective by Rundblad and Annaz (2010). They compared performance on metonymy to metaphor performance in ASD and typical children. While metaphor is considered to represent a mapping between two distinct conceptual domains, metonymy is a mapping within the same conceptual domain (Lakoff, 1987), and, as such, may be considered a less demanding. The study included picture stories with lexicalized metaphors and lexicalized metonymies incorporated in brief stories. The authors established developmental trajectories for each group, and for each task, first assessing performance on the two tasks relative to chronological age. While for the typical group performance on both metonymy and metaphor increased reliably with chronological age, no reliable correlation was found between scores and chronological age on either task in the ASD children. In this group, in addition, children performed significantly worse on the metaphor task. These two tasks also revealed two different trajectories. While for metonymy there was a development, for metaphor, performance was constant across time and ages, indicating what the authors label a zero trajectory. Furthermore, this study shows that vocabulary scores predict reliably metonymy in the ASD group, with improved performance with higher verbal age, while no similar relationship was found for metaphor comprehension. Thus, compared to typical controls, the ASD group displays a similar rate of development for their level of receptive vocabulary in the area of metonymy, whereas the difference in metaphor comprehension is significantly different. This study adds an important developmental perspective, suggesting that metaphors are an area of specific difficulty where development is not only delayed (as with metonymy), but also highly atypical and, most likely, compromised.

Gold et al. (2010) studied metaphor comprehension in Asperger syndrome in adolescent and adult participants using four types of expressions, free (literal) expressions (*pearl necklace*), conventional metaphors (*sealed lips*), novel metaphors (*firm words*), and nonsensical expressions (*violin tiger*). Their main goal was to establish the accuracy of interpreting such expressions and the degree of cognitive load involved, as measured by reaction times and brain activation. The results of this study showed that compared to typical controls, Asperger individuals present with problems, as reflected in significantly longer reaction times compared to typical controls. Furthermore, different patterns of activation, as seen in the N400 amplitude, were found between the Asperger participants and the control group, which reached significance for the category conventional and novel metaphor. While, in the control group conventional metaphors elicited least negativity, for the Asperger group, it was literal expressions. This suggests greater effort in the processing of metaphor across the board in ASD individuals, including even conventional metaphors, which can be stored, as well as novel metaphorical expressions. Moreover, in the Asperger group reaction times were significantly longer for the processing of nonsense expressions. The latter result is indicative of a specific problem evident in other studies of figurative language in autism, namely the inability to assess the plausibility of linguistic expressions events or facts (Tager-Flusberg, 1981; Paul et al., 1988).

An interesting study provides evidence of a specific dissociation between the processing of visually presented metaphors and verbally presented ones in autistic populations (Mashal and Kasirer, 2012). This study compared ASD and Learning Deficit children to typically developing controls. They used 11 subtests, ranging from figurative language interpretation including visual metaphors, idioms, conventional metaphors, novel metaphors, to homophones and semantic tests (synonymy, similarity). The authors analysed the data using a Principal Component Analysis to investigate for clustering of performance results. Results loaded on three different factors in all three groups in the study, and while there was a significant overlap in the loading between groups, both deficit groups displayed a clustering of all three verbal figurative language skills (idioms, conventional and novel metaphors) in the same factor, suggestive of the specific problems in that population. Moreover, in those two groups there was a dissociation between metaphors presented visually, and those presented verbally, as reflected in the results loading on two different factors. In contrast, the typical children displayed an association between idioms and conventional metaphors, which can be expected, given the nature of conventionalized expressions and idioms, and an association between visual and novel metaphors, which loaded together on a separate factor. This latter result suggests an integration in typical individuals of the processing of metaphors, irrespective of their modality (visual or verbal), most probably through a common underlying cognitive mechanism. This does not appear to be the case in the autism group and the group with learning deficits.

All of the clinical studies reviewed here document a dissociation between literal and figurative language in autism, which argues against a simple continuity model. Clearly a revision of this model is called for in the face of these data.

### Accounts of the pragmatic deficit: a specific deficit or not?

From the studies reviewed above and earlier findings, it becomes evident that there is a pervasive problem in the autistic spectrum in the broader domain of pragmatic aspects of language. However, there is a debate concerning the causes of this problem and what aspects of the autistic profile can account for the pragmatic deficit. One assumption is that the pragmatic deficit is not special and does not dissociate from the rest of language competence in autism. The idea is that performance on pragmatic tasks and the ability to process (ambiguous) language in context correlates directly with structural language competence (Norbury, 2005; Brock et al., 2008; Gernsbacher and Pripas-Kapit, 2012).

Alternatively, the pragmatic deficit can be linked to other traits in the autistic profile. Thus, one of the most widely accepted theories of what is causing the deficit in the domain of figurative language and metaphors, in particular, is based on Happé’s study and hypothesis that the deficit is caused by impaired mentalising skills and in terms of impaired ToM (see also Baron-Cohen et al., 1985; Happé, 1993; Baron-Cohen, 2000, 2001). Clearly, the ToM hypothesis can explain one aspect of what is necessary to be able to perceive others’ intentions, including those expressed verbally. Yet, many studies reveal increased problems with decrease in the transparency of the mapping between language structure and (intended) interpretation (MacKay and Shaw, 2004). All studies document a specific problem in the area of metaphors, even compared to closely related, but less demanding, phenomena, such as e.g., metonymy. This indicates that reading intentions (mentalising) needs to be operationalized accordingly on a finer scale of gradience, explaining difficulties and/or success in all types of figurative expressions.

A host of hypotheses attempts an account in terms of more general cognitive mechanisms dedicated to information processing. Some authors attribute the deficit to more general problems in executive functioning and the inability to suppress unnecessary information (Ozonoff et al., 1991; Mashal and Kasirer, 2012). This account links to the well-observed problem in assessing event plausibility (Tager-Flusberg, 1981), but also to the Weak Central Coherence account (Frith, 1989; Frith and Happé, 1994; Happé and Frith, 2006). Happé and Frith (2006) suggest that individuals with ASD have difficulties to understand metaphors, because they have a deficit in executive function and central coherence. This can be attributed to the fact that individuals with ASD display a bias for processing information locally rather than globally. Frith (1989) points out that in order to be able to understand a word or an expression they should be put in a concrete context. Context is even more important for figurative expressions, in order to process the intended meaning, rather than just the literal one. In fact, weak central coherence has been attributed as the source of pragmatic problems in individuals with ASD (Noens and van Berckelaer-Onnes, 2005). In addition, Norbury and Bishop (2002) found that people with ASD have difficulties in contextual integration, and the more ambiguous the expression is, the greater the problem in this population (Happé, 1997; Jolliffe and Baron-Cohen, 1999, 2000; López and Leekam, 2003; Brock et al., 2008).

Other accounts seek to explain the pragmatic deficit at the neural level in terms of a right hemisphere (RH) deficit (Gold and Faust, 2010; Gold et al., 2010). In one study, participants were asked to perform a semantic judgment task. The results indicated much less Right Hemispheric contribution to novel metaphor comprehension in ASD. Impaired RH activity was further documented in other studies of figurative language processing (Faust and Mashal, 2007).

Alternatively, it can be assumed that the inability to process figurative language arises from problems in information integration, especially when information is to be retrieved from multiple sources (e.g., problems with processing in context), and linking this to the more general deficit at global processing (top down) at the expense of enhanced local processing (bottom up). Of special interest here is that the well-documented problems in processing ambiguous information arise only in the context of language contra visual information (López and Leekam, 2003), and dissociates from structural language skills (Vulchanova et al., 2012a,b). Furthermore, there is evidence that visual and linguistic metaphors dissociate only in autistic participants, but not in typical children (Rundblad and Annaz, 2010). Therefore, it would be logical to conclude that the difficulties that people with autism demonstrate in figurative language are probably due to inability to either access both modalities at the same time or integrate information from more than one modality at the same time. While the visual context may assist interpretation in typical populations, it may create additional problems in deficit populations such as individuals with autism (Chahboun et al., in preparation).

Indeed, one of the main symptoms of ASD is the lack of information integration and absence of adaptability to the environment (Minshew et al., 1997; Brock et al., 2008). Many authors attribute this to the inability to gather together information in order to be able to distinguish between relevant and irrelevant information, in part attributable to weak central coherence (Frith, 1989; Happé and Frith, 2006; Vulchanova et al., 2012b). Selecting relevant features of the metaphor vehicle concept and suppressing the irrelevant ones has been suggested as the basic mechanism in metaphor comprehension (Rubio Fernandez, 2007).

It is widely argued that individuals with ASD are impaired in processing ambiguous linguistic information in context (López and Leekam, 2003; Brock et al., 2008). In addition, they often fail to attach context to their memories and are specifically impaired in processing social aspects of contextual information (Greimel et al., 2012).

Happé (1996) suggests that difficulties in global processing could be due to conceptual semantic deficits, but also to a failure in extracting perceptual properties from context. López and Leekam (2003) provide evidence that the ability to use context is spared in the visual domain, but reduced in the verbal one. Further, they document increased problems with increased complexity of the verbal stimuli and with higher level of ambiguity. This points to the limitations in the ability to use contextual information in individuals with ASD, but not a complete absence of this skill.

The extent to which individuals with autism can use context in disambiguation is an open question, and findings are controversial. Some authors consider that people with ASD are unable to use contextual information in sentence-processing tasks. Others still, claim that success or failure depend on the nature of the context: the more general the information provided by the context is, the more difficult it is for autistic participants to disambiguate homographs (Hermelin and O’Connor, 1967; Frith and Snowling, 1983; López and Leekam, 2003).

Saldaña and Frith (2007) and Tirado (2013) document that children with ASD have a normal reduction in reading times for expressions which are congruent with previous events, suggesting a relative strength at detecting congruence. It has also been argued that the ability to use context depends on structural language skills, and only ASD participants with poor language skills fail to use visual context (Norbury, 2005; Brock et al., 2008). What is clear from these studies is that different types of context present different processing demands, and autistic performance varies accordingly.

## Summary and conclusions

Language is a complex multi-layered and multifaceted system. In order to interpret language appropriately, users need a number of skills. Figurative language can be even more demanding in terms of processing. It is acquired relatively late and has a complex nature, which makes it even more difficult for atypical population, such as individuals with ASD, to understand. What skills are deemed necessary for language processing and figurative language, in particular? Adequate structural language competence, adequate semantic competencies and skills and vocabulary size (Norbury, 2004, 2005; Oakhill and Cain, 2012), inferencing skills; a developed conceptual system and a knowledge base (Schneider et al., 1989; Fuchs et al., 2012; Oakhill and Cain, 2012); information integration skills (context; evaluating plausibility and suppressing irrelevant information, Rubio Fernandez, 2007); mentalising and understanding intentions (see Kintsch, 2000 for a computer simulation model). Needless to say, many of these skills co-vary with language (e.g., semantic skills and vocabulary, conceptual knowledge, and the knowledge base are often directly associated with linguistic labels), so studying them in isolation and controlling for their impact on figurative language depends on the kind of measure adopted. Impairment in any one of these areas is sufficient to cause problems in the comprehension of figurative language. For instance, in order to understand one of the most demanding instances of figurative language, metaphor, the user not only needs to have prior experience and knowledge of the concepts that are being associated in a metaphorical expression, but also knowledge of their respective domains and the networks they form with other concepts in these domains (Keil, 1986; Bambini et al., 2011). This requires information integration and processing skills, beyond those required for simple concept combination (Barsalou, 1999; Wu and Barsalou, 2009), and depends on the ability to form associations, analogies and other top-down skills. If we take high-functioning autistic individuals as a test case, the cause of the persistent difficulty in figurative language becomes more evident. In this population, structural language is intact; they present with adequate semantic and conceptual skills, are good at compositional operations at the level of the sentence, perform adequately at a number of inferencing tasks (Tirado, 2013 PhD thesis), and usually pass first-order, and often second-order ToM tasks, and have an age-appropriate knowledge base, as attested by normal IQ scores. The only area where problems persist is information integration and inability to use information from the database adequately: evaluating plausible/implausible events; assessing what is relevant; combining information arising from different modalities.

Building on the original proposal by Kintsch (1998), an influential account of (reading) comprehension suggests that success at language processing depends on creating appropriate situation models. This means that the language user needs to create a mental representation of what the message is about, not what the message says (Zwaan, 1999). Based on the evidence in research on problems in the domain of figurative language interpretation, it is highly likely that autistic individuals have problems in building and making use of appropriate situation models. The models they build could in some respects be incomplete. More importantly, they might not be able to make use of them to understand the co-locutor’s intention with the message. It seems as though, they possess the necessary knowledge base, but cannot use it adequately, since they cannot judge plausibility (Tager-Flusberg, 1981; Paul et al., 1988), often fail at certain types of inferences, and are not always good at exploiting contextual information. It has been shown that typical children benefit from visual support and are better at processing visually presented metaphors (Epstein and Gamlin, 1994). However, multi-modal and multi-sensory information appears to be a problem in autism, despite intact visual processing *per se* (López and Leekam, 2003; Chahboun et al., in preparation). As a consequence, individuals in the autistic spectrum fail to integrate a situation model that integrates the necessary information, the speaker’s intent and the rest of the context in which all this must be used.

## Directions for future research

Most of the studies reviewed in this paper are heterogeneous and difficult to compare. They have used different methodologies, test skills in different figurative language domains, and often use largely heterogeneous groups of participants. Thus, quite often the range of participants is from mid-childhood to adulthood. Since one of the intriguing questions in research in developmental deficits is whether one can expect development in the comprehension of the different types of figurative expressions, more homogenous groups are required, in both longitudinal and cross-sectional studies (cf. Melogno et al., 2012b for a similar point). Similarly, the types of expressions selected in those studies vary tremendously, especially those that have been chosen as exponents of the target category. For instance, the degree to which expressions fall in the category of conventional metaphor needs to be tested prior to including it in an experiment. Likewise, other linguistic properties of the stimuli are important: frequency of expression and/or constituent words will affect processing; collocational frequency of the constituents in the expression (e.g., “buckle” and “button” are by far the most frequent complement fillers of “fasten”, as in fasten a buckle/a belt, so these phrases tells us little about argument structure competence in typical and deficit populations alike).

The observed dissociation between figurative (non-literal) and literal language processing in ASD lends support to findings about the neural correlates of idiomatic language processing in typical adult populations (Lauro et al., 2008), suggesting a bilateral involvement of fronto-temporal areas for idioms against selective activation of left inferior parietal areas in the case of literal expressions. The recruitment of the prefrontal cortex may reflect an active selection between alternative meanings when idioms are processed. This offers a new perspective for future research comparing the neural and cognitive mechanisms involved in figurative language comprehension in autism and typical populations.

Another intriguing line of research are recent accounts of the role of embodiment in human cognition and, specifically, in language comprehension (Barsalou, 2008). It has been suggested that the well-attested communication problems in autism could be partially driven by core (low-level) cognitive mechanisms, such as deficits in temporal coordination and sensori-motor impairment (e.g., motor movement). This type of account is consistent with models of embodied cognition in typical populations and is worth pursuing in future research (Eigsti, 2013).

An interesting, yet unexplored perspective are parallel studies of similar pragmatic deficits observed in different developmental disorders. For instance, Lacroix et al. (2010) document problems in idiom comprehension in French speaking children and adolescents with William’s syndrome. Similar results have been found while testing the ability to understand metaphors and sarcasm (Karmiloff-Smith et al., 1995; Annaz et al., 2009). Since WS is characterized by a relative strength in language and social interest, but poor conversational skills, contra impaired spatial cognition, it would be interesting to test how this population compares to the autistic spectrum, especially the higher end, where structural language is spared, too. Even more intriguingly, it has been suggested that the observed figurative language problems in WS may be attributed to poor semantic integration (Hsu, 2013).

Finally, if we are right in attributing the figurative language deficit to poor information integration and impaired situation models, appropriate tasks need to be set up to test for exactly these types of skills. Developmental deficits offer a rare glimpse into the, sometimes subtle, dissociations between and within cognitive domains, such as e.g., structural vs. extended (figurative) language, and as such, can shed light on how metaphors and other figurative expressions are processed in typical individuals, what kinds of demands this processing requires and at what cost. Future research should seek to provide a consistent comprehensive account of the mechanisms involved in language comprehension at the neural and cognitive levels in both typical and deficit populations (Dilkina and Lambon Ralph, 2013).

## Conflict of interest statement

The authors declare that the research was conducted in the absence of any commercial or financial relationships that could be construed as a potential conflict of interest.
